# Comparative Genomic Analyses of *Streptococcus pseudopneumoniae* Provide Insight into Virulence and Commensalism Dynamics

**DOI:** 10.1371/journal.pone.0065670

**Published:** 2013-06-19

**Authors:** Dea Shahinas, Christina S. Thornton, Gurdip Singh Tamber, Gitanjali Arya, Andrew Wong, Frances B. Jamieson, Jennifer H. Ma, David C. Alexander, Donald E. Low, Dylan R. Pillai

**Affiliations:** 1 Laboratory Medicine and Pathobiology, University of Toronto, Toronto, Canada; 2 Department of Microbiology, Immunology and Infectious Diseases, University of Calgary, Calgary, Canada; 3 Public Health Ontario, Toronto, Canada; 4 DNA Core Facility, Public Health Ontario, Toronto, Canada; 5 Pathology and Laboratory Medicine, University of Calgary, Calgary, Canada; University of Kansas Medical Center, United States of America

## Abstract

*Streptococcus pseudopneumoniae* (SPPN) is a recently described species of the viridans group streptococci (VGS). Although the pathogenic potential of *S. pseudopneumoniae* remains uncertain, it is most commonly isolated from patients with underlying medical conditions, such as chronic obstructive pulmonary disease. *S. pseudopneumoniae* can be distinguished from the closely related species, *S. pneumoniae* and *S. mitis*, by phenotypic characteristics, including optochin resistance in the presence of 5% CO_2_, bile insolubility, and the lack of the pneumococcal capsule. Previously, we reported the draft genome sequence of *S. pseudopneumoniae* IS7493, a clinical isolate obtained from an immunocompromised patient with documented pneumonia. Here, we use comparative genomics approaches to identify similarities and key differences between *S. pseudopneumoniae* IS7493, *S. pneumoniae* and *S. mitis*. The genome structure of *S. pseudopneumoniae* IS7493 is most closely related to that of *S. pneumoniae* R6, but several recombination events are evident. Analysis of gene content reveals numerous unique features that distinguish *S. pseudopneumoniae* from other streptococci. The presence of loci for competence, iron transport, pneumolysin production and antimicrobial resistance reinforce the phylogenetic position of *S. pseudopneumoniae* as an intermediate species between *S. pneumoniae* and *S. mitis*. Additionally, the presence of several virulence factors and antibiotic resistance mechanisms suggest the potential of this commensal species to become pathogenic or to contribute to increasing antibiotic resistance levels seen among the VGS.

## Introduction

Accurate differentiation between pneumococci and other viridans group streptococci (VGS) is essential given their different clinical manifestation. *S. pneumoniae* is the causative agent of several diseases, including sepsis, abscess formations and meningitis. In contrast, while the other members of the VGS are benign commensals, they have been implicated in infective endocarditis and infections in patients with neutropenia [1]. *S. pneumoniae* is phenotypically similar to the several commensal species: *S. mitis*, *S. oralis*, *S. infantis*, and *S. tigurinus*, subsequently often causing problems of identification in clinical microbiology laboratories [2], [3]. This complexity of classification has been further compounded by the introduction of *S. pseudopneumoniae* (SPPN), which is a recently designated species belonging to the VGS, falling directly within the Mitis 16S rRNA classification. Despite having >99% 16s rRNA gene identity with *Streptococcus pneumoniae* and *Streptococcus mitis, S. pseudopneumoniae* exhibits DNA-DNA hybridization values of <70% and is phenotypically distinct [4]. Unlike *S. pneumoniae*, *S. pseudopneumoniae* is optochin resistant in the presence of 5% CO_2_, bile insoluble and lacks the pneumococcal capsule. However, *S. pseudopneumoniae* is optochin susceptible under ambient atmosphere, often causing difficulty in distinguishing it from *S. pneumoniae* in diagnostic laboratories [4]. *S. pseudopneumoniae* has also demonstrated greater resistance levels to several antimicrobial agents than typeable pneumococci [5].

The phenotypic features that characterize *S. pseudopneumoniae* are consistent among strains that have been historically classified as “atypical” or “unusual” streptococci, such as hemolysis patterns on blood agar media [6]. *S. pseudopneumoniae* isolates have been isolated from respiratory cultures from patients with lower respiratory tract disease and have been associated with chronic obstructive pulmonary disease (COPD) exacerbation. Pathogenicity of the species has also been well described in a murine model of infection [7]. Additionally, *S. pseudopneumoniae* has been isolated from cystic fibrosis patients and has been associated with symptoms of pneumonia, bronchitis, and chronic sinusitis [8]. However, the pathogenic potential and the underlying genetic identity of *S. pseudopneumoniae* is not well characterized in relation to the overt pathogen *S. pneumoniae* or the more avirulent *S. mitis* [2], [7], [9].

Members of the Mitis group are naturally transformable as reflected by a high degree of variability between *S. pneumoniae* clones at the genomic level and by the production of well-characterized competence pheromones and pheromone receptors [2]. In order to establish genetic differences between *S. pneumoniae*, *S. mitis* and *S. pseudopneumoniae*, analysis at the genomic level is necessary to tease out both overt and subtle divergence between the three species. To date, there is no complete genome of *S. pseudopneumoniae* and as such, we sought to sequence the genome of an isolate for comparative genomic analysis. To define genomic differences and establish genetic targets for clear identification of these closely related organisms, whole genome shotgun sequencing of a representative *S. pseudopneumoniae* patient isolate, IS7493, and initial assembly were performed with the Genome Sequencer FLX (Roche, Basel, Switzerland). Isolate IS7493 was obtained from the sputum of a patient with human immunodeficiency virus (HIV) who had documented pneumonia. The objectives of this study included comparative genomic analysis to the closely related members of the Mitis group *S. pneumoniae* and *S. mitis*, as well as broad whole genome comparisons to other streptococci. In particular, our focus was on known virulence and antibiotic resistant factors that may be relevant to *S. pseudopneumoniae* within a clinical context. Our findings suggest that *S. pseudopneumoniae* IS7493 is an intermediate isolate between *S. pneumoniae* and *S. mitis* and is lacking several known virulence factors such as the choline binding proteins PcpA, PspA, PspC, the capsule biosynthesis genes and the *piaABCD* iron transport encoding locus. Hence, *S. pseudopneumoniae* is genetically more akin to a commensal member of the VGS. However, the presence of several virulence genes, as well as a transposable element containing antibiotic resistance factors suggest that this species has the capability to be pathogenic.

## Materials and Methods

### Source of clinical isolates

Four isolates of *S. pseudopneumoniae* were received at the Public Health Ontario Laboratories (PHOL), Ontario, Canada in the period 2007–2009. These isolates were characterized as “atypical” streptococci on the basis of growth characteristics, optochin resistance in the presence of 5% CO_2_ and bile insolubility. The patient population from which the isolates are derived are summarized in Table 1. Retrospective analysis resulted in the classification of these streptococci as *S. pseudopneumoniae* based on close resemblance with the published phenotypic features of this newly designated species [4], namely optochin resistance in the presence of 5% CO_2_, bile insolubility and absence of the capsule locus. IS7493, an isolate from an endotracheal tube aspirate in a septic HIV-positive patient was selected for whole genome sequencing. *In vitro* susceptibility testing and interpretation was performed by broth microdilution according to Clinical and Laboratory Standards Institute guidelines for *S. pneumoniae* (non-meningitis breakpoints) [10].

**Table 1:** Patient age, gender and source of isolates used in this study.

Isolate Number	Age	Gender	Sample
IS7493	47	M	Endotracheal tube
IS4938	77	M	Bronchial washing
PH9143	67	M	Bronchial washing
IS2502	68	F	Sputum

### DNA preparation

Total genomic DNA extraction was performed with QIAamp DNA Mini kit following the manufacturer's protocol (Qiagen, Germantown, MD) with a few modifications. Briefly, bacteria were propagated from a single colony in 10 plates for 16 hours and then resuspended in 1 mL phosphate buffered saline (PBS pH 7.4). One QIAamp column was used per each plate. After centrifugation, the bacteria were resuspended in 180 µL of ATL buffer, followed by the addition of proteinase K (Qiagen, Germantown, MD). The mixture was incubated at 56°C for 30 minutes. Subsequently, 400 µg of RNase A (Qiagen, Germantown, MD) were added and incubated at room temperature for 2 minutes. The rest of the steps were followed as per manufacturer's protocol with elution volume of 50 µL 1× Tris-EDTA (TE) for every column.

### Sequencing, assembly and annotation of the *S. pseudopneumoniae* IS7493 genome

Shotgun sequencing of the *S. pseudopneumoniae* IS7493 genome was performed by GS-FLX pyrosequencing with Titanium chemistry. Filter-pass sequence reads (331,392) were assembled using the gsAssembler (Roche, Basel, Switzerland) into 123 total contigs (333 bp to 161,538 bp, N50 = 62,237) with an average of 50× coverage. MAUVE [11], [12] was used to align contigs against available streptococci reference genomes including *S. pneumoniae* R6 and *S. mitis* NCTC12261. Contig order was verified by PCR and gaps were filled by Sanger sequencing of PCR amplicons, which were labeled using the Life Technologies BigDye Terminator v3.1 Cycle Sequencing kit (Applied Biosystems, Foster City, CA) and sequenced via capillary electrophoresis on an Applied Biosystems 3130*xl* DNA Analyzer (Foster City, CA). RAST server [13] and the NCBI Prokaryotic Genome Automatic Annotation Pipeline (PGAAP; http://www.ncbi.nlm.nih.gov/genomes/static/Pipeline.html) were used for initial annotation of the *S. pseudopneumoniae* IS7493 genome. Automated annotation was followed by manual inspection. Notably, the ORFs described in this manuscript were subjected to detailed manual review. The GC content and GC bias were determined using the Artemis Comparison Tool (ACT) [14]. For the pDRPIS7493 plasmid, the functional domain identity and hypothetical protein prediction was achieved using the conserved domain and protein prediction databases of the National Center for Biotechnology Information (NCBI) and Protein Databank (PDB).

### Comparative genome analysis

The assembled and annotated genome of *S. pseudopneumoniae* IS7493 was compared to all the available genomes of streptococci in the SEED Server both at the sequence and functional level [15]. Dot plot analysis comparison was done with the top hit of the comparative genome analysis (*S. pneumoniae* R6) and the closely related *S. mitis* NCTC12261. The whole genome phylogenetic analysis was conducted using the CVTree platform [16].

### *lytA* and *comC* gene amplification and sequencing

To verify the presence of locus SPPN_05425 in all isolates (copy 1 of *lytA* which contains the V317R polymorphism) as well as to perform bidirectional Sanger sequencing of this locus in all isolates, the following primers and PCR conditions were used as described previously: Forward: 5′-ATCCTAGCCAAGAACGCAGT-3′ Reverse: 5′-TCCCAAATTCCTCCTACTCG-3′ [17]. The amplified product covers the region from 101 bp–1434 bp of 1020413–1021969 in the *S. pseudopneumoniae* IS7493 genome.

To verify the presence of locus SPPN_09835 (copy 2 of *lytA* which contains the Thr290-Gly291 deletion) in all isolates as well as to perform bidirectional Sanger sequencing of this locus in all isolates, the following primers were used: Forward 5′-AAGCTTTTTAGTCTGGGGTG-3′ and Reverse 5′-AAGCTTTTTCAAGACCTAATAATATG-3′ [18]. For amplification of both loci, the *Pfx* polymerase (Life Technologies, Carlsbad, CA) was used at an annealing temperature of 57°C (60 seconds) and elongation temperature of 68°C (90 seconds). The amplified products were purified using the Qiagen PCR product cleanup kit (Qiagen, Germantown, MD) before Sanger sequencing.

For the amplification and sequencing of *comC* (SPPN_11375), the following primers were used: Forward 5′-CAATAACCGTCCCAAATCCA-3′ Reverse 5′-AAAAAGTACACTTTGGGAGAAAAA-3′ [19]. In this case, the annealing temperature was 55°C and conditions were kept as described previously [20]. Pherotype classification was followed as previously described [21]. All multiple sequence alignments were generated using MUSCLE [22], [23].

### Nucleotide sequence accession number

The whole genome sequence and the plasmid sequence of *S. pseudopneumoniae* IS7493 were deposited in the DDBJ/EMBL/GenBank databases under the accession numbers, CP002925 and CP002926, respectively.

## Results and Discussion

### Genome sequence of *S. pseudopneumoniae* IS7493: general features

The clinical relevance of *S. pneumoniae* has been well characterized. Additionally, *S. mitis* and other members of the VGS have increasingly gained notoriety in clinical onset and have been associated with several manifestations. The ability to speciate prokaryotic organisms genetically has increased with the current advances in molecular identification technology. Our group published the first full genome of a clinical isolate of *S. pseudopneumoniae* [24] and here, we provide comparative genomic analysis to the overt pathogen, *S. pneumoniae*, and a relatively benign commensal, *S. mitis*.

**Figure 1:**
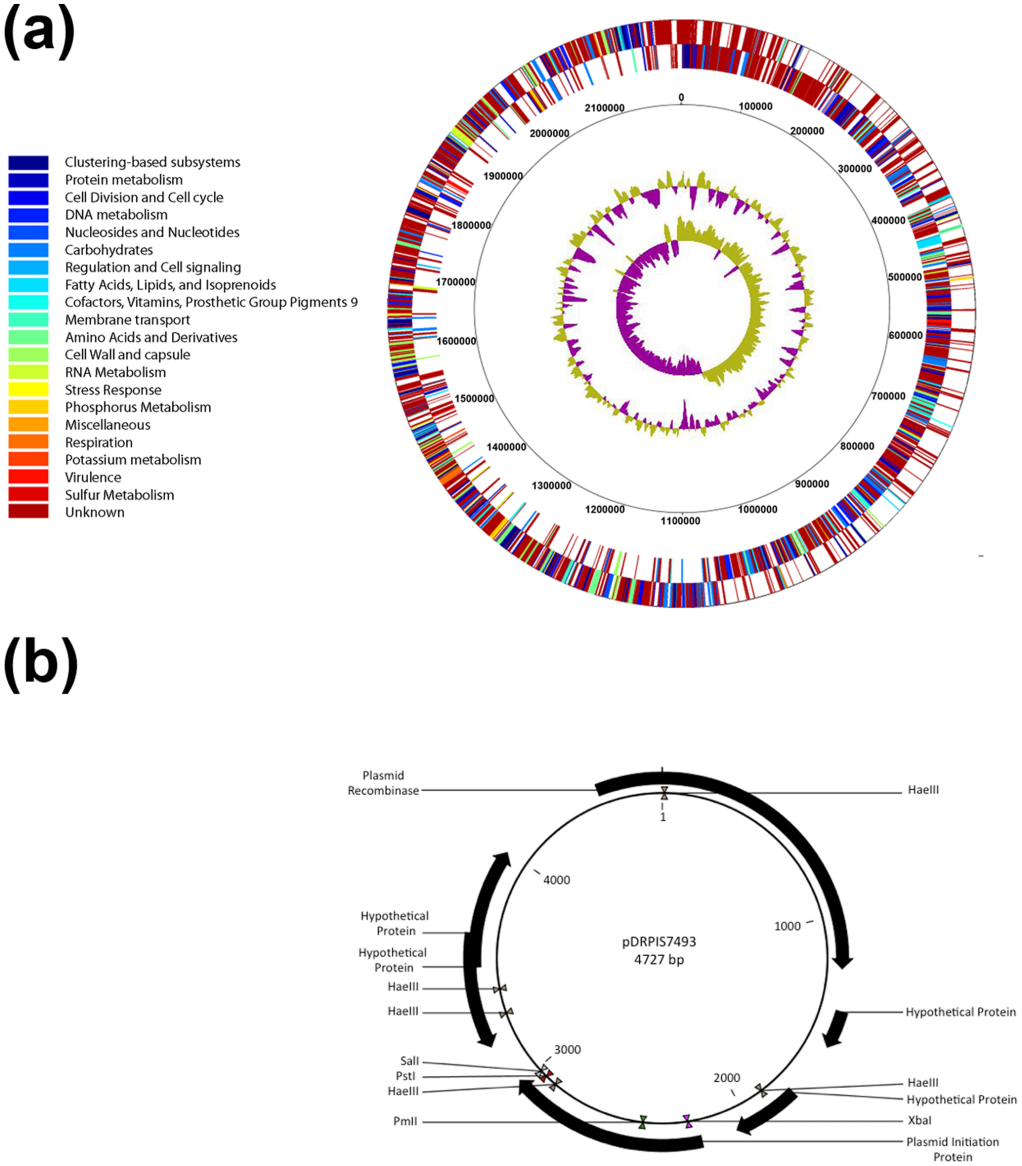
Overview of the genome of *S.* *pseudopneumoniae* IS7493. (a) Representation of the *S. pseudopneumoniae* IS7493 circular genome. The inner circles represent GC content (outer) and GC bias (innermost). (b) Full assembly revealed the presence of plasmid pDRPIS7493.

General features of the *S. pseudopneumoniae* IS7493 genome and assembly statistics are listed in Table S1. In addition to a single circular (2,190,731 bp; 39.8% GC content) chromosomal genome (Figure 1a), isolate IS7493 also harbors a single plasmid of 4.7 kb (38.3% GC content), which was conventionally assigned as pDRPIS7493 (Figure 1b).

**Figure 2:**
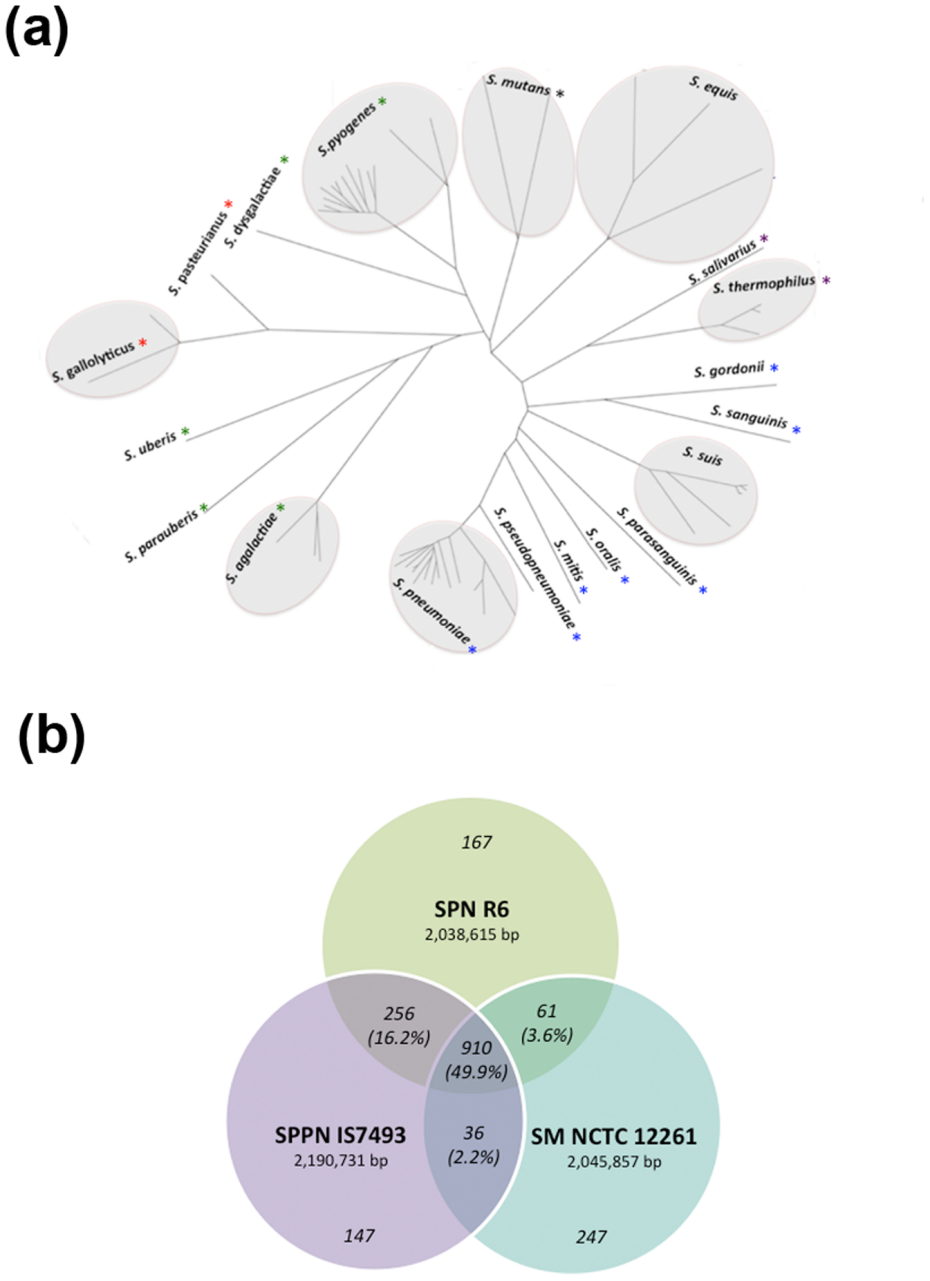
Comparative genome analysis of *S .pseudopneumoniae* IS7493 with the selected closely related streptococci. (s) Whole genome phylogenetic tree of selected VGS isolates (Neighbor joining) generated using CVTree [16]. The branch lengths are representative of phylogenetic distance. Color legend for asterisks: blue, Mitis group; black, Mutans group; purple, Thermophilus group; green, Pyogenic group; and red, Bovis group. (b) Summary statistics for the genome comparison of *S. pseudopneumoniae* IS7493 with the closely related *S. pneumoniae* R6 and *S.mitis* NCTC12261. The CDS that are not accounted for in this comparison coded for hypothetical proteins.

Pairwise genome alignment with available genomes of *Streptococcus* species revealed that the genome of *S. pseudopneumoniae* IS7493 is most closely related to the genome of *S. pneumoniae* R6 (File S1). Neighbour joining whole genome phylogenetic analysis with selected members from the *Streptococcus* genus (Figure 2a) also showed that at the genetic level, *S. pseudopneumoniae* IS7493 is an intermediate species falling between *S. mitis* and *S. pneumoniae*, but with closer proximity and presumed relativity to pneumococci. In keeping with these results, dot plot analysis comparison of *S. pseudopneumoniae* IS7493 with *S. pneumoniae* R6 and *S. mitis* NCTC12261 genome sequences indicated differences in genetic content between the three species and implicated *S. pseudopneumoniae* IS7493 as a closer relative of *S. pneumoniae* R6 than *S. mitis* NCTC12261 (Figure S1).

Genome annotation of the open reading frames based on RAST and PGAAP showed that the chromosome of *S. pseudopneumoniae* IS7493 contains 2,230 coding sequences (CDS), 41 tRNA and 3 rRNA operons, which is comparable in size to completed *S. pneumoniae* and *S. mitis* genomes (2.04–2.24 Mb) [25]. There are 910 CDS common between *S. pseudopneumoniae* IS7493, *S. pneumoniae* R6 and *S. mitis* NCTC12261 (Figure 2b). *S. pseudopneumoniae* IS7493 has 256 CDS in common with *S. pneumoniae* R6 that are not present in *S. mitis* NCTC12261. In contrast, there are only 36 CDS common between *S. mitis* NCTC12261 and *S. pseudopneumoniae* IS7493 that are not present in *S. pneumoniae* R6. The CDS that are not accounted for in this comparison coded for hypothetical proteins. A complete list of all CDS as well as the functional and sequence comparisons with *S. pneumoniae* R6 and *S. mitis* NCTC12261 has been tabulated in File S2. The plasmid, pDRPIS7493, contains six CDS: a plasmid replication protein, plasmid mobilization protein, plasmid recombination enzyme, and three hypothetical protein sequences (Figure 1b). Sequence comparison and domain search showed that pDRPIS7493 is most similar with plasmids pSMQ172 (NCBI: NC_004958) and pER13 (NCBI: NC_002776) from *S. thermophilus*. Comparison of pDRPIS7493 with the rolling circle replicating plasmid pDP1 in pneumococci showed no significant homology.

The alignment between *S. pneumoniae* R6 and *S. pseudopneumoniae* IS7493 is conserved in ten regions interspersed by segments of symmetrical inversion. However, these rearrangements do not affect the preferred location of coding sequences on the leading strand (Figure 1a). The near ten-fold increase in shared CDS with *S. pneumoniae* than *S. mitis* suggests that this species originated likely from *S. pneumoniae*. Recombination events that may have lead to *S. pseudopneumoniae* are likely complex and difficult to tease out, compounded heavily by the ability of the VGS to undergo horizontal gene transfer. In particular, *S. pseudopneumoniae* IS7493 originated from the lower respiratory tract of an immunocompromised patient with pneumonia. Given that members of the VGS, particularly within the Mitis group, readily colonize the upper respiratory tract as a conglomerate known as the oropharyngeal flora, it is possible that both the environmental niche and the clinical onset may have contributed to the acquisition and/or loss of several virulence factors seen in the genome of *S. pseudopneumoniae*. In addition, the acquisition of a plasmid outside of the Mitis group indicates the level of VGS diversity found within this particular isolate, within the lower respiratory tract and suggests complex ecological interactions.

*S.pseudopneumoniae* IS7493 harbors several mobile and repeat elements that have been previously characterized among VGS. Among these elements are 41 insertion sequences (IS) previously described in other streptococci (File S3). The known IS in the closely related *S.pneumoniae* and *S.mitis* are: IS*1381*, IS*Spn2* and the newly described IS*Smi3* and IS*Smi1* transposase [25]. The genome was also scanned for intergenic repeat elements Repeat Unit of Pneumococcus (RUP), and *Streptococcus pneumoniae* Rho-Independent Terminator-like Element (SPRITE) and BOX which are present among *S.pneumoniae* genomes and indicate ancestral mobility of these elements for the purpose of gene regulation [26]. *S.pseudopneumoniae* contains 22 RUP A, 2 RUP B1, 3 RUP B2, no RUP C, 77 BOX A, no BOX B, 95 BOX C and no SPRITE repeat elements. It has been proposed that RUP elements may still be mobile [27]. Together with the abundance of repetitive elements in general in *S.pseudopneumoniae* IS7493, these findings suggest that this genome is under active selection pressure and subject to a high rate of horizontal transfer events. RUP elements were not found to be abundant in *S.mitis* B6 [25] and most likely represent the close relationship of *S.pseudopneumoniae* IS7493 to the highly recombinant *S.pneumoniae* strains.

### Features of the *S. pseudopneumoniae* IS7493 accessory genome and virulence factors

Members of the Mitis group are characterized by mosaic genes as a result of interspecies gene transfer and recombination events, as observed for the virulence genes encoding neuraminidase A and IgA protease [28], [29]. As a consequence, *S. pneumoniae* and *S. mitis* contain large accessory genomes. For instance, the accessory genome of *S. mitis* B6 has been estimated to constitute over 40% of all coding sequences within its genome [25].

One major feature of the already characterized accessory genome of the Mitis group is an array of cell surface proteins, which are essential in mediating interactions with host cells. These cell surface proteins are classified into two groups: choline binding proteins (CBPs) [30] and cell wall bound proteins containing the characteristic LPXTG motif [31]. Several pneumococcal specific virulence factors have been characterized among these proteins such as the choline binding proteins PspC (CbpA), PspA and PcpA, hyaluronidase HylA and a genomic island that contains *ply* plus *lytA* encoding the potent cytolysin pneumolysin and the major autolysin, *lytA*. *S. pseudopneumoniae* IS7493 contains several of the factors listed above, most notably the latter two virulence genes. *S. pneumoniae* and *S. mitis* have been shown to contain large accessory genomes, in relation to the predominant core genome. As a result, assessment of the Mitis group accessory genome constituents in *S. pseudopneumoniae* IS7493 was conducted. It was found that *S. pseudopneumoniae* IS7493 harbors the coding sequences for the potent cytolysin, pneumolysin (locus SPPN_09795) and autolysin *lytA* (2 copies: SPPN_05425 and SPPN_ 09835), but is lacking the pneumococcal surface proteins, PspC and PspA and in addition, only contains a truncated remnant of PcpA (Table 2). Similar to what has been previously described for *S. mitis* B6, *S. pseudopneumoniae* IS7493 also contains homologues of *licD1*, *licD2* and *licD3*, which are known to play an important role in choline decoration of teichoic acids [25]. The presence of *licD* homologues presents evidence that choline-containing teichoic acids are present in *S. pseudopneumoniae* [25]. Consistent with this observation, the genome of *S. pseudopneumoniae* IS7493 encodes for 20 choline-binding proteins. Choline- binding proteins are anchored to the cell wall by hydrophobic interactions with choline-containing teichoic acids and are composed of a choline-binding module consisting of 20-mer repeats of amino acids and a non-conserved functional domain [30]. From the 20 CBP coding sequences of *S. pseudopneumoniae* IS7493, 18 contain the classical 20-mer repeats. All six CBPs that are implicated in cell separation and murein hydrolysis in *S. pneumoniae* are present in all four *S. pseudopneumoniae* isolates included in this study: LytB, LytC, Pce, CbpD, CbpF and LytA (Table 2) [30], [32]. Both the presence of CBPs and homologies of *licD1*, *licD2* and *licD3*, which are known to play an important role in choline decoration of teichoic acids, present evidence that choline-containing teichoic acids are present in *S. pseudopneumoniae* [25]. CBP genes are polymorphic and diversification of these genes by duplication of repeat modules and recombination events has been previously reported as a key factor in the organization of the Mitis group [30].

**Table 2:** CBPs in *S. pseudopneumoniae* IS7493, *S. mitis* and *S. pneumoniae*.

SPPN IS7493 Locus & Gene	Sequence Identity (%)	Species		
		SM B6	SM NCTC12261	SPN R6
0050 *cbp8*	76 (B6)	+	−	−
00605 *cbp4*	97 (B6)	+	+	−
00665 *cbpI*	52 (B6)	+	+	−
00670 *cbp8*	71 (B6)	+	−	−
00945 *cbp5*	77 (B6)	+	−	truncated
01925 *cbp14*	62 (B6)	+	−	−
02235 *cbpG*	61 (R6)	−	−	truncated
02240 *cbpF*	74 (R6)	+	+	+
02265 *cbpJ/cbp9*	72 (B6)	+	−	−
02595 *cbp6*	84 (B6)	+	−	−
03180 *cbpI*	63 (B6)	+	+	−
05425 *lytA*	88 (R6)	+	+	+
06615 *cbpE/pce1*	95 (R6)	+	+	+
06645 *cbpI*	49 (B6)	+	+	−
06650 *cbp8*	66 (B6)	+	−	−
07200 *lytB*	89 (B6)	+	+	+
07725 *lytC*	89 (R6)	+	+	+
09140 *pcp^a^*-truncated	51 (R6)	−	−	+
09740 *cbp5*- truncated	90 (B6)	+	−	truncated
09835 *lytA*	86 (R6)	+	+	+
09875 *pcpA*- truncated	87 (R6)	−	−	+
10050 *cbpG*	57 (R6)	−	−	truncated
10055 *cbpJ/F/pcpC*	66 (B6)	+	+	+
10220 *cbp*	75 (R6)	−	−	+
11215 *cbpD*	93 (B6)	+	+	+

In *S. pneumoniae*, several LPXTG proteins that are part of the accessory genome are linked to pathogenicity [33]. Our analysis showed that the *S. pseudopneumoniae* IS7493 genome contains nine proteins with the LPXTG cell wall attachment motif (Table 3). Five of these proteins have higher sequence identity to the *S. pneumoniae* R6 homologues, one is 90% identical to an LPXTG domain protein from *S. mitis* SK564 and one novel protein contains no homologues and can only be characterized as a member of this family on the basis of the presence of the LPXTG domain. Remarkably, the four clinical isolates of *S. pseudopneumoniae* isolates do not contain the gene that encodes IgA1 protease. However, a gene encoding neuraminidase (A) NanA is present in *S. pseudopneumoniae* IS7493, but is truncated due to a frameshift mutation. While CBPs and LPXTG proteins are monocistronic, NanA is part of a metabolic operon [34], which is conserved in the genome of *S. pseudopneumoniae* IS7493 apart from the truncation of the NanA gene (Table 4).

**Table 3:** Cell wall anchor proteins in *S. pseudopneumoniae* IS7493.

Annotation	Species			
	SM B6	SM NCTC12261	SPN R6	SPPN IS7493
Hypothetical protein, 152mer repeat	+	+	+	0905 (68% R6)
Endo-beta-N-acetylglucosaminidase	+	+	+	02930 (91% R6)
Sialidase A (neuraminidase A), *nanA*	+	−	+	-
Cell wall-associated serine proteinase precursor, *prtA*	+	−	+	03355 (90% R6)
Hypothetical protein, coiled-coil domain; KA-rich 77mer repeats	+	−	−	05815 (66% B6)
Hypothetical protein, Pro-rich; interspersed repetitive domains (95mers)	+	+	−	-
Surface anchored proteins	+	+	−	-
Hypothetical protein, Pro-rich;36mer repeat	+	+	−	-
Serine protease	+	−	−	04145 (96% B6)
Zinc metalloprotease, *zmpB*	+	+	+	-
Glycine rich protein (87mer repeat)	+	−	+	-
Beta-galactosidase, *bgaA*	+	−	+	0395 (96% R6)
N-acetyl-beta-hexosaminidase	+	−	−	-
LPXTG-motif cell wall anchor domain-containing protein	−	−	−	0710
Cell wall surface anchor family protein, Ser rich *monX*	+	−	−	-
Alkaline amylopullulanase, *pula*	+	+	+	02085 (94% R6)
LPXTG-motif cell wall anchor domain-containing protein	−	−	−	07060

**Table 4:** Structure of the neuraminidase operon in *S. pseudopneumoniae* IS7493.

Annotation in *S.pneumoniae*	Species	
	SM B6	SPPN IS7493
Regulator	+	+
Hypothetical protein	+	+
N-acetylmannosamine kinase	+	+
N-acetylneuraminate lyase	+	+
Hypothetical protein	+	+
Hypothetical protein	−	+
Hypothetical protein	+	+
*satA* ABC transporter permease	+	+
*satB* ABC transporter permease	+	+
*satC* ABC transporter substrate binding protein	+	+
PTS system, IIBC components	−	+
NanE, ManAC-6P 2-epimerase	+	+
Oxidoreductase	−	+
NanB neuraminidase	−	+
ABC transporter permease	−	+
ABC transporter permease	−	+
ABC transporter substrate-binding protein	−	+
Hypothetical protein	−	+
NanA neuraminidase	+	truncated
Acetyl xylan esterase	+	+

Annotation of the genes in the operon has been followed as per Gualdi *et al*. [34].

The presence of the glycolytic enzymes enolase and GAPDH, however, was evident in these isolates. As well, a gene encoding another major accessory LPXTG motif protein, the Ser-rich “monster” gene (*monX*) was absent in these isolates. However, even though the *monX* is absent from the two closely related reference genomes *S. pneumoniae* R6 and *S. mitis* NCTC12261, it is present along with its associated genes in the genome of *S. mitis* B6 [25]. Furthermore, the genomic region within *S. pseudopneumoniae* IS7493 that contains *monX* is also present in *S. gordonii* where it encodes the protein named GspB and has been associated with infective endocarditis [35]. This suggests that these virulence genes are part of the common accessible genomic material of naturally transformable streptococci and can be acquired via horizontal gene transfer. Since the *nanA* sequences of oral streptococci cluster closely together, it has been suggested that this is an indication of frequent genetic exchange at this locus [28], [36].

Even though the absence of an IgA protease homologous gene in *S. pseudopneumoniae* is surprising because its presence has been shown in some of *S. mitis* and most of *S. pneumoniae* strains, these genes may be subject to genomic loss. Since *S. pseudopneumoniae* harbors no capsule, it is possible that there may be no fitness cost associated with the loss of this gene via recombination events in the genome. In contrast, the truncation of the neuroaminidase *nanA* gene may be disadvantageous for host cell attachment owning to the concept that loss of this gene or vaccination with NanA protein impairs pneumococcal persistence in the nasopharynx and otitis media in a chinchilla infection model [37], [38]. NanA serves as a sialidase in these mucus rich environments and absence may rationalize the association of *S. pseudopneumoniae* infections with COPD, which together with the absence of the capsule and pili biosynthesis genes, may suggest that *S. pseudopneumoniae* infections are both specialized and localized. The presence of the glycolytic enzymes enolase and GAPDH further corroborates this postulation, because these enzymes bind human plasminogen (PLG), which is converted to the protease plasmin (PA) and promotes degradation of various ECM compounds [39]. The plasminogen activator receptor (uPAR) levels are upregulated in COPD patients [40], suggesting that the binding of plasminogen by these enzymes is important for COPD progression. In addition, it is known that non-capsulated pneumococci adhere better to epithelial cells [40]. As such, improved adherence of *S. pseudopneumoniae* to epithelial cells is a feature that would be clinically important for progression of emphysema in COPD patients.

Several virulence factors can be found in *S. pneumoniae* genomes. The commensal *S. mitis* also contains a majority of the characterized pneumococcus virulence factors, owing to the fact that they are crucial for colonization and adherence to host cells [25]. Apart from the repertoire of genes described above for the choline decoration of the cell wall, *S. pseudopneumoniae* IS7493 also contains the following factors that have been implicated for their importance in interaction with host cells: the zinc metalloprotease ZmpB, the serine protease HtrA, the surface protein PavA, enolase, GAPDH, hemolysin HlyIII, the CBPs CbpF, LytB, LytC and Pce, the oligopeptide transporters AmiA, AliA, AliB and the manganese transporter PsaA.

Two-component system (TCS) regulatory proteins that consist of a histidine kinase and a response regulator play a significant role in the regulation of virulence in *S. pneumoniae*. *S. pseudopneumoniae* IS7493 contains 12 of the 13 TCS that have been described in *S. pneumoniae* including the phosphate regulating TCS04/PhoRP [41]. All 12 TCS of the *S. pseudopneumoniae* IS7493 have sequence identities >95% with their pneumococcal homologues. In comparison, *S. mitis* B6 is missing both TCS04 and TCS06, but contains the phosphate transport system PhoU [25]. *S. pseudopneumoniae* IS7493 contains both TCS04 and the PhoU transport system, but is missing TCS06. TCS06 is involved in the regulation of the important virulence factor CbpA [42]. As such, the absence of the TCS06 regulatory system is consistent with the absence of the gene encoding CbpA. In addition, *S. pseudopneumoniae* IS7493 lacks the *rlrA* islet, which serves as a pilus encoding cluster [43]. In contrast to *S. mitis*, which lacks both iron uptake systems *piu/pia* [25], *S. pseudopneumoniae* strains contain the *piu* operon in addition to the siderophore twin-arginine transport (TAT) iron uptake system *tatA/C*, which is found in *S. mitis*. The *S. pseudopneumoniae* isolates lack the *pia*ABCD operon, which is encoded in the pneumococcus pathogenicity island. Though this *tatA/C* system has not been implicated in virulence within VGS, TAT excreted proteins are important virulence factors in both *Pseudomonas* and *Yersinia* [44], [45]. *S. pseudopneumoniae* isolates in this study lack the *pia*ABCD operon. However, both piaABCD and piuABCD iron transport systems are not required for pathogenicity. In pulmonary and systemic models of pneumococcal infection, non-synonymous mutations in either *piu* or *pia* had minor impact on virulence, but virulence was severely attenuated in strains that contained mutations in both *pia* and *piu* operons [46], [47]. Analysis of the multitude of virulence factors within *S. pseudopneumoniae* IS7493 and corresponding clinical isolates does suggest the possibility of causing disease.

A defining feature of the species is that *S. pseudopneumoniae* is not encapsulated. *S. pseudopneumoniae* IS7493 has the antimicrobial resistance associated genes *pbp1a*, *aliA* and *pbp2x* adjacent to the *S. pneumoniae* capsule locus, but does not have any of the capsule biosynthesis genes. This isolate is penicillin resistant for the meningitis breakpoint, but not for the non-meningitis breakpoints according to the guidelines of the Clinical and Laboratory Standards Institute (CLSI) [10]. *S. mitis* B6 has the genes necessary for the regulation of the capsule polysaccharide but not the genes for the synthesis of the capsule polysaccharides [25]. The capsule biosynthesis genes have been shown to be located between a set of transposases in *S. pneumoniae*. In line with absence of coding sequences in genetically labile regions, the *S. pseudopneumoniae* isolates of this study are also missing the *S. pneumoniae* hyaluronidase *hylA* gene, which is encoded in an island flanked by an IS200-like element and consisting of many sugar metabolism component genes in addition to the *hylA* gene. As described for the closely related *S. mitis*, in *S. pseudopneumoniae* IS7493, all these genes are also missing. Hyaluronidase breaks down hyaluronic acid, a component of connective tissue, but its precise role in pathogenicity has not been well elucidated [39].

Full genome assembly and annotation revealed that the pneumolysin gene is present in *S. pseudopneumoniae* IS7493 in a genetically labile region of the genome. The pseudopneumococcal pneumolysin gene *ply* (SPPN_09795) is located in proximity to one of the two *lytA* genes (SPPN_09835) as previously described for *S. pneumoniae* [17], and is flanked by several recombinases and regulatory proteins. The sequence of SPPN_09795 is 99% identical to the sequence of the *ply* gene of *S. pneumoniae* R6. LytA is responsible for the characteristic lysis that results in the deoxycholate (bile) soluble phenotype in all human pneumococcal isolates [17], [18]. A two amino acid deletion (Thr290-Gly291 in choline binding repeat 6) is responsible for the inhibitory effect of deoxycholate on the enzymatic activity of lytic amidases from “atypical” pneumococci [17]. Pneumococci previously referred to as “atypical” due to their bile insolubility, optochin resistance and lack of capsule are now being classified as *S. pseudopneumoniae*, polymorphisms in the *lytA* gene have been exploited as a molecular tool for the quick clinical differentiation of these isolates [18].

**Figure 3:**
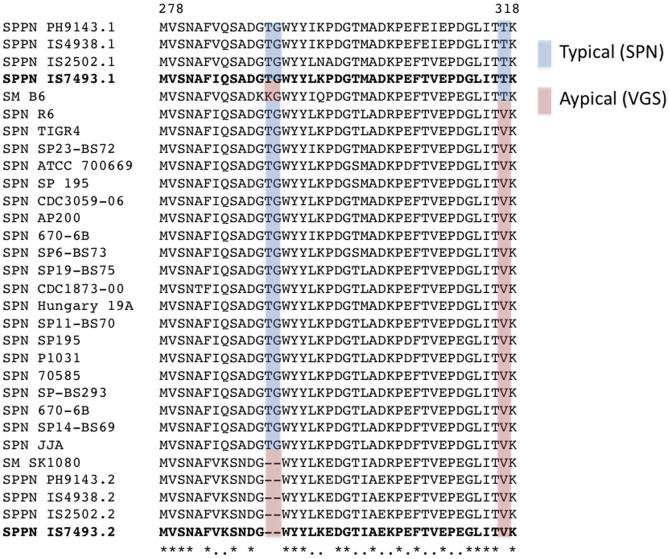
*S.* *pseudopneumoniae* cholin binding domain protein sequence of autolysin (LytA). VGS LytA proteins have been classified into typical and atypical categories based on residue 317 and presence/absence of Thr290-Gly291. Blue: functional amidase in presence of bile; Red: non-functional amidase in presence of bile; SM: *S. mitis*; SPN: *S. pneumoniae*; SPPN: *S. pseudopneumoniae*. Four SPPN clinical isolates are included. LytA alleles (.1&.2) refer to loci SPPN_05425 and SPPN_09835, respectively. The multiple sequence alignment was generated with the Multiple Sequence Comparison by Log- Expectation (MUSCLE) algorithm [22], [23]. * denote sequence identity and . denote sequence similarity at the bottom of the figure. In bold: LytA protein sequences of *S. pseudopneumoniae* IS7493.

As alluded to above, whole genome sequencing analysis revealed the presence of two *lytA* alleles in *S. pseudopneumoniae* IS7493 at the loci SPPN_05425 and SPPN_09835. SPPN_05425 appears to encode a typical LytA protein with no Thr290-Gly291 deletion but with threonine present at position 317, while SPPN_09835 appears to encode for an atypical LytA protein carrying a deletion at position 290–291 and valine at position 317. Multiple sequence alignment of LytA protein sequences from the 4 clinical isolates, *S. pseudopneumoniae* IS7493, *S. pneumoniae* and *S. mitis* sequences available in GenBank showed that all 4 *S. pseudopneumoniae* isolates carry both copies of the *lytA* gene: one typical *lytA* and one atypical *lytA* (Figure 3). Perhaps these mutations (Val317Thr and Thr290-Gly291 deletion) contribute to the phenotype in addition to other factors, such as those involved in the allolysis of these bacteria [25].

### Antibiotic resistance molecular mechanisms

Antibiotic resistance trends among the VGS are a growing issue within clinical settings. In particular, attention is being put towards the Mitis group, predominantly due to the ability of these organisms to readily uptake antibiotic resistance factors. The susceptibility profile of each of the isolates of this study consisting of the minimum inhibitory concentrations (MICs) for the pneumococcus panel of antibiotics is summarized in Table 5.

**Table 5:** Summary of the MICs for the *S. pseudopneumoniae* isolates of this study with interpretation based on CLSI breakpoints for *S. pneumoniae*.

Antibiotic	IS7493	PH9143	IS4938	IS2502
Amoxicillin/Clavulanic acid	≤2 S	≤2 S	4 I	≤2 S
Azithromycin	>2 R	≤0.25 S	≤0.25 S	≤0.25 S
Cefepime	1 S	1 S	2 I	≤0.5 S
Cefotaxime	0.5 S	0.25 S	0.25 S	0.12 S
Ceftriaxone	0.5 S	0.25 S	0.25 S	0.12 S
Cefuroxime	2 R	1 I	1 I	≤0.5 S
Chloramphenicol	4 S	2 S	2 S	≤1 S
Clindamycin	>1 R	>1 R	≤0.12 S	≤0.12 S
Daptomycin	0.12 NI	0.25 NI	0.5 NI	0.25 NI
Erythromycin	>2 R	≤0.25 S	≤0.25 S	≤0.25 S
Linezolid	0.5 S	0.5 S	1 S	1 S
Penicillin	1 S	2 R	4 I	0.03 S
Tetracycline	>8 R	≤1 S	≤1 S	≤1 S
Tigecycline	0.03 NI	0.06 NI	>0.12 NI	0.06 NI
Trimethoprim/Sulphamethoxazole	>4 R	2 I	>4 R	2 I
Vancomycin	≤0.5 S	≤0.5 S	≤0.5 S	≤0.5 S

All susceptibility interpretation shown as per non-meningitis pneumococcus CLSI breakpoints.

S: Susceptible; I: Intermediate resistant; R: Resistant; NI: Non-interpretable.

One of the key characteristics for classification of *S. pseudopneumoniae* is the demonstration of optochin resistance in the presence of 5% CO_2_. Optochin resistance has been associated with polymorphisms in the nucleotide sequence coding for the c subunit of the F_0_F_1_ ATP synthase [48]. The gene sequence of *S. pseudopneumoniae* IS7493 F_0_F_1_ ATP synthase subunit c showed a single non-synonymous substitution: phenylalanine to tyrosine substitution at codon 5 (Phe5Tyr). Previously reported mutations of this gene that have been associated with optochin resistance in pneumococci occur at different residues and thus this represents a novel mutation unique to *S. pseudopneumoniae* [48]. However, at this stage, we cannot conclude that this novel mutation confers optochin resistance in this species. Optochin resistance in the presence of CO_2_ and susceptibility in ambient conditions is a key phenotypic characteristic in *S. pseudopneumoniae* and targeting these mutations may be a valid diagnostic identification molecular method that may be applied to distinguish between other members of the Mitis group.

In addition to the substitution in F_0_F_1_ ATP synthase subunit c, *S. pseudopneumoniae* IS7493 and isolate 2 harbor the full sequence of integron Tn2010, found within multidrug resistant isolates of *S. pneumoniae*, and accounting for tetracycline and macrolide molecular mechanisms of resistance. Isolate 7493 is tetracycline and erythromycin resistant. Tn2010 constituent genes *ermB*, *mefA*, *tetM*, and *megA* are highly conserved amongst both pre- and post- poly conjugate vaccine multidrug resistant *S. pneumoniae* isolates [49]. The TetM gene is also present in *S. mitis* B6, but as part of Tn5801, which consists of only TetM copy and short flanking regions [25]. The tetracycline resistance determinant TetM is associated with the nested Tn916 transposable element in Tn2010 in most multiple antibiotic resistant isolates of *S. pneumoniae* such as Spain ^23F^ -1, MDR Canada 19A and MDR Canada 19F [49]–[51]. A study done with 140 *S. pseudopneumoniae* clinical isolates found high rates of decreased susceptibilities and resistance to erythromycin (57%) and tetracycline (43%), which may be accounted for by the acquisition of this transposable element from closely related species [8].

Consistent with previous reports of the VGS, the *S. pseudopneumoniae* isolates in this study also contain the aminoglycoside resistance associated genes: aminoglycoside 3′-phosphotransferase *aphA*, streptothricin acetyltransferase *sat* and aminoglycoside 6-adenylyl transferase *aadE*. These genes are not located in one single cluster as described in *Staphylococcus*. sp, *Enterococcus*. sp and *S. mitis* B6, but rather in disparate genomic regions as in most of the *S. pneumoniae* genomes [52].

Among the *S. pseudopneumoniae* IS7493 penicillin binding protein genes (PBP), the sequences of PBP2X, PBP2A and PBP2B are similar to the homologues in penicillin resistant *S. pneumoniae* with protein sequence identity ranging between 95–99%. *S. pseudopneumoniae* IS7493 PBP1B is 98% identical in protein sequence to PBP1B of *S. pneumoniae* R6.

*S. pseudopneumoniae* IS7493 PBP1A is more similar to the *S. mitis* homologues (89–91% protein sequence identity). In addition, since the two genes, *pbp1a* and *pbp2x*, flank the capsule locus, this finding points to a recombination event at this locus that may have resulted in the loss of the capsule biosynthesis genes. This postulation is consistent with the previous suggestion that recombination events of commensal members within the Mitis group have taken place by loss of virulence genes from pathogenic bacteria, rather than the evolution of pathogenic bacteria from commensal species [53]. Furthermore, *S. pseudopneumoniae* is placed within a unique position between *S. pneumoniae* and *S. mitis*, leading to confusion over whether the clinicians should use VGS or pneumococcus antibiotic breakpoint values for treatment.

### Competence and fratricide in *S. pseudopneumoniae*

**Figure 4:**
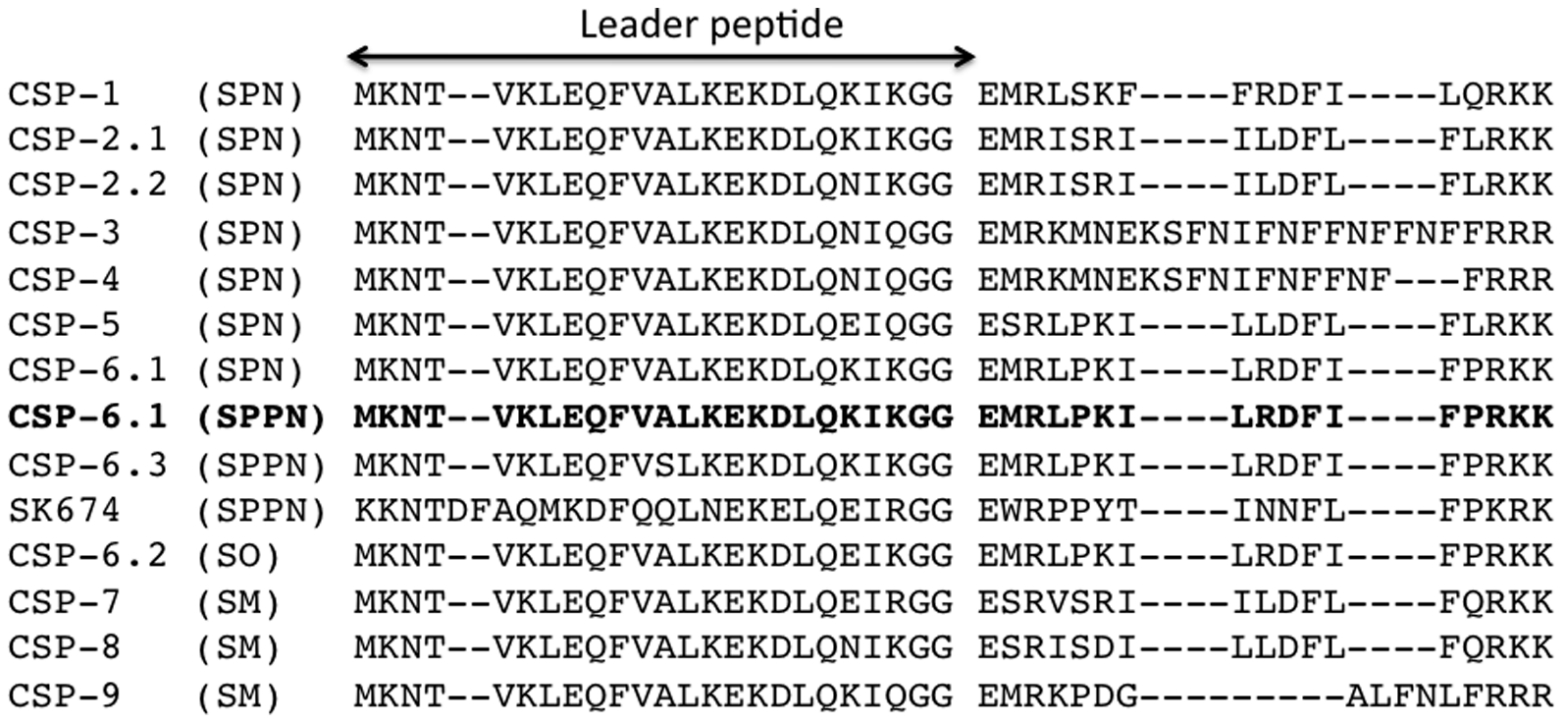
*S.* *pseudopneumoniae* competence stimulating peptide (CSP) sequences (leader and mature peptide region sequence is shown). According to the classification by Whatmore *et a*l. [21], *S. pseudopneumoniae* encodes a type 6.1 CSP. SM: *S. mitis*; SPN: *S. pneumoniae*; SPPN: *S. pseudopneumoniae*; SO: *S. oralis*. The multiple sequence alignment was generated with the Multiple Sequence Comparison by Log- Expectation (MUSCLE) algorithm [22], [23]. In bold: CSP sequences of *S. pseudopneumoniae* IS7493.

The divergence and genetic diversity seen in the Mitis group streptococci are thought to be primarily driven by horizontal gene transfer [54], [55]. In *S. pneumoniae*, induction of the competent state occurs when the extracellular concentration of the competence stimulating peptide (CSP) reaches a critical level and is sensed by the two component regulatory system ComDE, a histidine kinase receptor and a response regulator, encoded by *comD* and *comE*, respectively [56], [57]. Two allelic variants dominate amongst *S. pneumoniae*, *comC1* and *comC2*, resulting in two clinically dominating pherotypes producing CSP-1 and CSP-2, respectively [21], [58]. Induction of competence by CSP is restricted by ComD receptor recognition and therefore, among pneumococci, competence is typically restricted to occur within one pherotype [59]. The *comC* gene, encoding CSP, is polymorphic within the *pneumoniae-mitis-pseudopneumoniae* cluster [2]. According to a pherotype classification scheme introduced by Whatmore *et a*l. [21], the four *S. pseudopneumoniae* isolates encode a type 6.1 CSP, which has been previously observed among *S. pneumoniae* isolates, but not among *S. mitis* isolates (Figure 4). Apart from type 6.1 CSP, which is the most common pherotype observed for *S. pseudopneumoniae*, Leung *et al*. found two more pherotypes associated with *S. pseudopneumoniae*, CSP6.3 and SK674 [20]. A distinct signature sequence in the CSP leader peptide has been observed for each of the phylogenetic clusters [2]. The *S. pseudopneumoniae* IS7493 genome contains all the genes that make up the competence machinery apart from the late competence protein ComGE, the function of which remains unclear. In addition, *S. pseudopneumoniae* IS7493 harbors the gene that encodes the immunity factor ComM, thought to protect competent cells from their own lysins [60].

The detection of the competence operon within the *S. pseudopneumoniae* genome, responsible for DNA uptake, suggests a fully operational system in order to uptake divergent species genomic material. This finding is not surprising, given the competent nature of the VGS but whether *S. pseudopneumoniae* is naturally competent remains to be tested. Apart from the use of the competence operon as a potential identification diagnostic tool to differentiate between other VGS, a distinct signature sequence in the CSP leader peptide has been observed for each of the phylogenetic clusters as shown in figure 4 and as previously published [2]. In the absence of a capsule, classification by pherotype may present an attractive approach for Mitis group streptococci. This suggestion is further supported by published data that *S. pneumoniae* isolates sharing the same serotype have a high probability of belonging to the same pherotype [61]. In addition, the use of *comC* sequences was found to isolate a *S. pseudopneumoniae* cluster distinct from other oral streptococci [20]. With the introduction of two additional CSPs to the *S. pseudopneumoniae* repertoire, further study is needed to identify divergent CSPs and to assess if “cross-talk” between varying CSPs may occur within *S. pseudopneumoniae* strains or even other species in the Mitis group [20]. However, a limitation of using pherotype classification is that pherotype is a clonal property and may vary independently of the serotype, especially amongst those isolates lacking a capsule.

**Figure 5:**
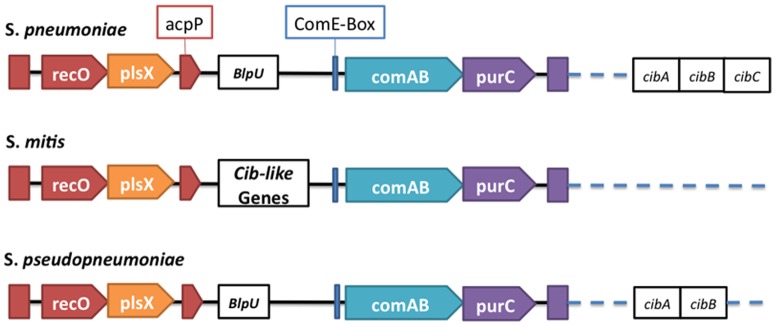
Summary of the genetic architecture of the competence locus that distinguish three homologous competence systems from one-another. Blp: Bacteriocin like peptide. Cib: Competence induced bacteriocin.

Other mechanisms that may influence competition during colonization have also been proposed. Blp bacteriocins are small antimicrobial peptides with a two component regulatory system similar to CSP and confer inter-species competition [62]. In *S. pseudopneumoniae* IS7493, the full *blp* cluster is well conserved and contains the two-component system BlpRS, the pheromone ABC transporter region as well as the genes that encode BlpX, BlpY and BlpZ. A second cluster of bacteriocins upstream of *comAB* also exists as has been described for Mitis group streptococci [25]. This cluster contains distinguishing features among the bacteria of this group (Figure 5). It includes genes that encode competence induced bacteriocins/immunity proteins. The bacteriocin gene *blpU* is present in both *S. pneumoniae* R6 and *S. pseudopneumoniae* IS7493. In addition, *S. pneumoniae* R6 contains the competence regulated genes *cibAB* at this locus, the coding sequence for the immunity factor CibC and the coding sequence for bacteriocin like protein U (BlpU) located 5′ of the comAB cluster. *S. pseudopneumoniae* IS7493 also contains the *cibAB* genes at this locus as well as the *BlpU* gene, but not the *cibC* gene. *cibABC* is absent in *S. mitis* NCTC12261, but the full operon is present in *S. mitis* B6 [25] suggesting that this system may have arisen prior to the onset of divergent species. CibAB is essential for allolysis, but is insufficient for induction of allolysis on its own [63]. It triggers downstream effects and allolysis via activation of CbpD, LytA and LytC, resulting in lysis and the release of DNA [63]. The CibC immunity factor protects cells from CibAB induced allolysis [63]. The absence of this gene from *S. pseudopneumoniae* IS7493 suggests that it may also serve as a genetic reservoir for other closely related bacteria.

## Conclusion

The genome of *S. pseudopneumoniae* IS7493 is a typical example of genomic rearrangement within a complex community of bacterial organisms. In addition, it can be seen that successful gene acquisition and/or gene loss has occurred within this species, evidenced by the larger genome in comparison to the closely related reference genomes of *S. pneumoniae* R6 and *S. mitis* NCTC12261. *S. pseudopneumoniae* may be of pathogenic potential and may act as source or recipient of DNA in recombination events generating new alleles under high selective pressure such as antibiotic treatment, for instance. As it stands, the genome of *S. pseudopneumoniae* IS7493 contains an elaborate set of cell surface proteins, several markers of antibiotic resistance and a large number of well-characterized virulence factors derived from *S. pneumoniae*, which may not be necessarily directly involved in virulence, but may be necessary for the interaction of these bacteria with host cells. Nonetheless, the presence of the full competence machinery suggests that these bacteria have the potential to serve as a genetic reservoir as well as to become pathogenic in human disease. The intricate molecular knowledge of the *S. pseudopneumoniae* genome presented here will undoubtedly lead to further targets for identification within the Mitis group and provide further discriminatory power in clinical settings. *S. pseudopneumoniae* may be thought of as an organism that readily treads the fine balance between pathogen and commensal with the necessary machinery and capability to facilitate both roles in a complex ecological niche.

### Ethics statement

As this is a surveillance and bacterial strain characaterization study conducted at the Public Health Laboratory, this work did not obtain formal ethics approval from an IRB. No patient identifying information is included and the study was retrospective in nature. As such, no patient consent was obtained.

## Supporting Information

Figure S1 Dot plot analysis comparison of the genome alignment of *S. pseudopneumoniae* IS7493 with the closely related *S. pneumoniae* R6 and *S. mitis* NCTC12261.
(PDF)

File S1 List of closely related streptococcal species based on genome comparison with *S. pseudopneumoniae* IS7493.
(XLS)

File S2 Detailed genome comparison analysis at the functional and sequence level between *S. pseudopneumoniae* IS7493, *S. pneumoniae* R6 and *S. mitis* NCTC12261.
(XLS)

File S3 A list of the insertion sequence (IS) elements identified in the genome of *S.pseudopneumoniae* IS7493.
(PDF)

Table S1 Table of assembly statistics for genome sequencing.
(PDF)
