# Supplementary figures and images for: Comparative Genomic Analyses of Streptococcus pseudopneumoniae Provide Insight into Virulence and Commensalism Dynamics
Source: PLoS One. 2013 Jun 19;8(6):e65670. doi: 10.1371/journal.pone.0065670 (PMC3686770; doi:10.1371/journal.pone.0065670)

## *S. pneumoniae*

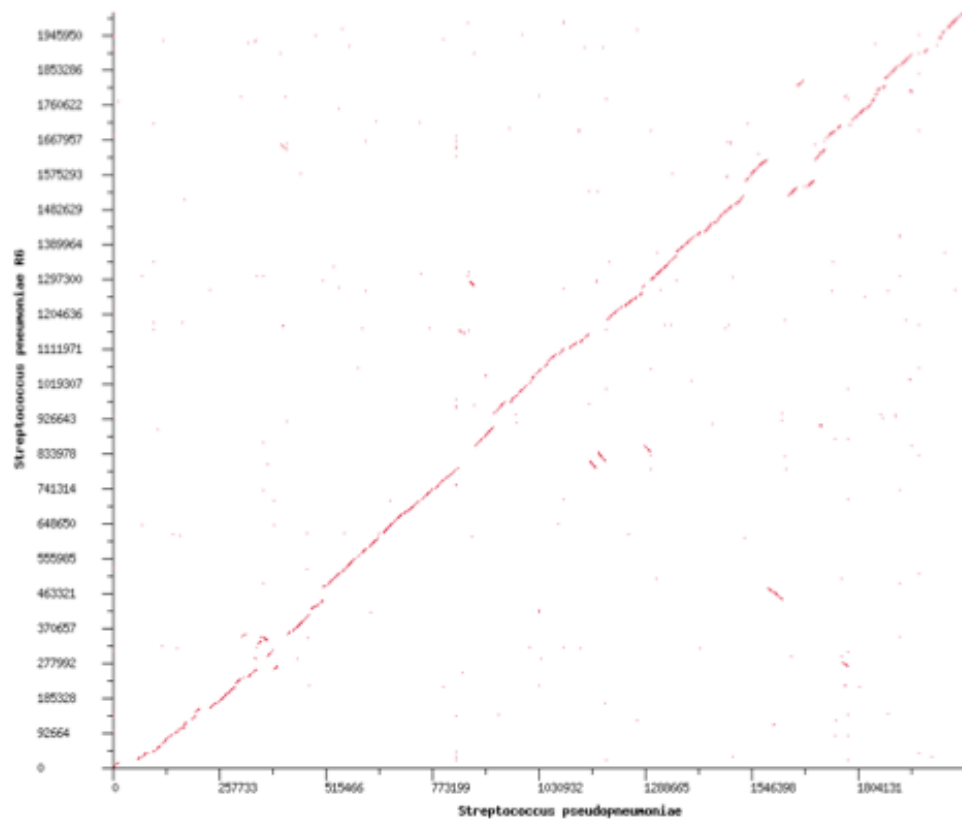

## *S. mitis*

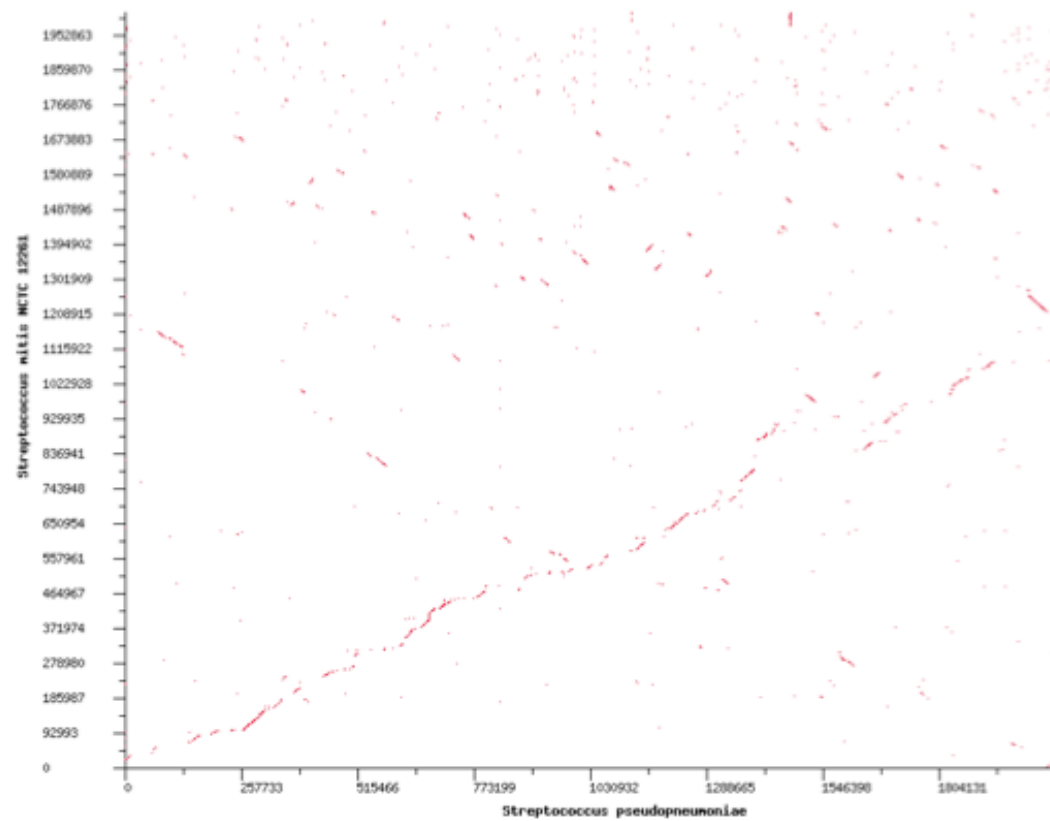

## *S.pseudopneumoniae*

Supplement: Figure S1 — Dot plot analysis comparison of the genome alignment of S. pseudopneumoniae IS7493 with the closely related S. pneumoniae R6 and S. mitis NCTC12261. (PDF) [file pone.0065670.s001.pdf]
