# Supplementary material for: Comparative Genomic Analyses of Streptococcus pseudopneumoniae Provide Insight into Virulence and Commensalism Dynamics
Source: PLoS One. 2013 Jun 19;8(6):e65670. doi: 10.1371/journal.pone.0065670 (PMC3686770; doi:10.1371/journal.pone.0065670)
Supplement: File S3 — A list of the insertion sequence (IS) elements identified in the genome of S.pseudopneumoniae IS7493. (PDF) [file pone.0065670.s004.pdf]

INSERTION SEQUENCES (IS) IDENTIFIED IN THE GENOME OF *STREPTOCOCCUS PSEUDOPNEUMONIAE*

The IS sequences were determined using IS Finder as per citation:

Siguier P. et al. (2006) ISfinder: the reference centre for bacterial insertion sequences. Nucleic Acids Res. 34: D32-D36 The database URL (http://www-is.biotoul.fr).

equences producing ignificant alignments	IS Family	Group	Origin	Score (bits)	E (value)
SSpn5	IS1380		*Streptococcus pneumoniae*	3255	0.0
SSpn9	IS30		*Streptococcus pneumoniae*	2313	0.0
SSmi3	IS30		*Streptococcus mitis*	2252	0.0
SSpn2	IS630		*Streptococcus pneumoniae*	1770	0.0
S630-Spn1	IS630	-	*Streptococcus pneumoniae*	1443	0.0
SSpn7	IS5	ISL2	*Streptococcus pneumoniae*	1427	0.0
S1381	IS5	ISL2	*Streptococcus pneumoniae*	1306	0.0
S1202	ISNCY	IS1202	*Streptococcus pneumoniae*	1047	0.0

S1381A	IS5	ISL2	*Streptococcus agalactiae*	876	0.0
S1167	ISL3	-	*Streptococcus pneumoniae*	813	0.0
S1167A	ISL3	-	*Streptococcus pneumoniae*	749	0.0
SSpn4	IS3		*Streptococcus pneumoniae*	731	0.0
SSg1	ISL3	-	*Streptococcus gordonii*	559	e-156
SSpn1	IS3	IS3	*Streptococcus pneumoniae*	529	e-147
SSpn10	IS110		*Streptococcus pneumoniae*	494	e-136
SSmu1	IS3	IS150	*Streptococcus mutans*	472	e-130
SSth6	IS3	IS150	*Streptococcus thermophilus*	422	e-115
SSmi1	IS30		*Streptococcus mitis*	329	7e-87
SSeq4	IS1634		*Streptococcus equi*	186	7e-44
SSpn8	IS30		*Streptococcus pneumoniae*	167	6e-38
SSsu3	IS630		*Streptococcus suis*	143	9e-31

SSeq6	IS30		*Streptococcus equi*	141	4e-30
SSeq3	IS3	IS150	*Streptococcus equi*	141	4e-30
SSth1	ISL3		*Streptococcus thermophilus*	135	2e-28
SSeq1	ISL3		*Streptococcus equi*	133	9e-28
S861	IS3	IS150	*Streptococcus agalactiae*	109	1e-20
S1191	IS256	-	*Streptococcus thermophilus*	105	2e-19
SSag9	IS30		*Streptococcus agalactiae*	103	8e-19
S905	IS256	-	*Lactococcus lactis*	98	5e-17
S1193D	ISL3	-	*Streptococcus thermophilus*	92	3e-15
S1193	ISL3	-	*Streptococcus thermophilus*	92	3e-15
SSeq2	ISNCY	IS1202	*Streptococcus equi*	90	1e-14
SStso1	IS630		*Streptococcus sobrinus*	86	2e-13
SSmu2	ISL3		*Streptococcus mutans*	76	2e-10

SPlu5	IS200/IS605	IS200	*Photorhabdus luminescens*	70	1e-08
SSmi2	IS1182		*Streptococcus mitis*	66	2e-07
SApl3	IS3	IS150	*Actinobacillus pleuropneumoniae*	62	3e-06
SSsu4	IS200/IS605	IS200	*Streptococcus suis*	56	2e-04
SSpn6	IS200/IS605	IS200	*Streptococcus pneumoniae*	56	2e-04
S1469	IS200/IS605	IS200	*Clostridium perfringens*	56	2e-04
SHar1	IS3	IS150	*Herminiimonas arsenicoxydans*	54	7e-04
