# Supplementary material for: Comparative Genomic Analyses of Streptococcus pseudopneumoniae Provide Insight into Virulence and Commensalism Dynamics
Source: PLoS One. 2013 Jun 19;8(6):e65670. doi: 10.1371/journal.pone.0065670 (PMC3686770; doi:10.1371/journal.pone.0065670)
Supplement: Table S1 — Table of assembly statistics for genome sequencing. (PDF) [file pone.0065670.s005.pdf]

Supplemental Table 1: Summary of the general features and assembly statistics of *S. pseudopneumoniae* IS7493 genome.

**Features of Genome**	
Sequencing	Shotgun Sequencing-Unpaired Reads
Average Read Length	333 bps
Number of Reads	331392
GC %	39.5%
**Assembly Statistics**	
Reference Genome	Pairwise Alignment- SPN R6
Assembly	De Novo (De Bruijin)
Average Coverage	54x
Number of Reads Not Assembled	5017
Number of contigs	1
Genome Size	2190731 bp
Annotation of Genome	RAST & PGAAP
**Other Features**	
Number of SNPs Relative to SPN R6	88879
